# CyberKnife radiotherapy for malignant fibrous histiocytoma of the chest wall: A case report and review of the literature

**DOI:** 10.3892/ol.2014.1995

**Published:** 2014-03-24

**Authors:** ZHEN WANG, XIN-HU WU, BING LI, QING-TAO KONG, ZE-TIAN SHEN, JING LI, ZHI-BING LIU, XI-XU ZHU

**Affiliations:** 1 Department of Radiation Oncology, Jinling Hospital, Nanjing University School of Medicine, Nanjing, Jiangsu 210002, P.R. China; 2 Department of Dermatology, Jinling Hospital, Nanjing University School of Medicine, Nanjing, Jiangsu 210002, P.R. China

**Keywords:** malignant fibrous histiocytoma, chest wall, CyberKnife

## Abstract

Malignant fibrous histiocytoma (MFH) is the most common type of soft tissue sarcoma, but rarely originates in the chest wall. Surgical resection is considered to be the most reliable treatment, however, no consensus has been reached concerning the best treatment for unresectable MFH. The current study presents the case of a 77-year-old male with MFH of the chest wall. The patient developed a painless mass and intermittent fever over a four-month period. A computed tomography scan demonstrated a large inhomogeneous lesion in the right chest wall, which was subsequently diagnosed via biopsy as a MFH. Since the tumor was an unresectable mass, CyberKnife^®^ radiotherapy was conducted. Following the treatment, a marked reduction in the tumor size was observed with a tolerable level of toxicity. The sequencing analysis also revealed an in-frame deletion (delE746-A750) in exon 19 of the epidermal growth factor receptor gene. Based on this result, gefitinib was administered to the patient at a dose of 250 mg/day.

## Introduction

Malignant fibrous histiocytoma (MFH) was first described as a distinct histological type of soft tissue sarcoma in 1964 (1). MFH may occur in the extremities, retroperitoneum and trunk, however, it rarely originates in the chest wall. Yoshida *et al* (2) reported that primary MFH of the chest wall predominantly affects elderly male patients with a variable clinical presentation.

Surgery is considered to be the most adequate therapy for MFH and resection with negative microscopic margins decreases the incidence of local recurrence (3). Therefore, the predominant *modus operandi* is wide excision, however, it may, lead to a considerably reduced quality of life. Radiotherapy (RT) has also been described as a definitive treatment for patients with MFH and unresectable or positive surgical margins (4). Furthermore, the advances in RT present stereotactic body RT (SBRT) as a novel technique for the treatment of human tumors. SBRT (for example CyberKnife^®^ RT) allows the delivery of a higher dose of radiation to the tumor while reducing the quantity of irradiation to the surrounding normal tissue (5). The current study reports the treatment of a patient with an unresectable MFH of the chest wall using CyberKnife^®^ RT.

## Case report

### Patient presentation and diagnosis

The current study presents a patient who developed an unresectable MFH in the chest wall. As the tumor was an unresectable mass, CyberKnife^®^ RT was conducted and, following the treatment, a marked reduction in the tumor size was observed with a tolerable level of toxicity. In addition, a separate literature search was performed using PubMed on February 1, 2012. The search criteria used were ‘malignant fibrous histiocytoma’ and ‘CyberKnife’, with the search ‘case reports’ used as a limitation; the search criteria included all languages. Review of the literature identified only one case of MFH with Cyberknife^®^ treatment and the tumor was reduced following Cyberknife^®^ treatment. Written informed consent was obtained from the patient for the publication of this case report and the accompanying images.

### Treatment and clinical course

**Figure 1:**
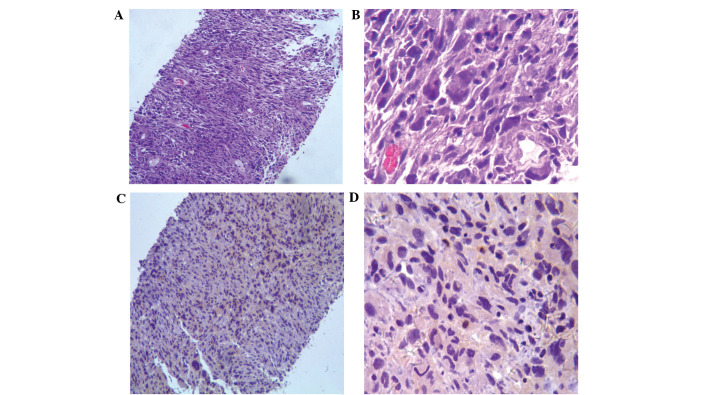
Pathological features. Histopathological examination revealed (A and B) the presence of pleomorphic spindle cells arranged in sheets and fascicles with a prominent storiform pattern (stain, hematoxylin and eosin; magnification, ×100 for A and ×400 for B) and (C and D) immunostaining for vimentin in the majority of tumor cells (stain, immunoperoxidase; magnification, ×100 for C and ×400 for D).

In August 2012, a 77-year-old male presented to the Jingling Hospital (Nanjing, China) with a four-month history of a gradually increasing painless mass in the right chest wall and intermittent fever. The patient’s medical history included prostatic hyperplasia, cholecystolithiasis and a cyst of the right kidney. The patient had no history of smoking or asbestos exposure. The tumor was elastic-hard, tender and characterized by a distinct border between the tumor and the normal tissue. The laboratory examinations showed mild anemia, hyponatremia, hypoalbuminemia and a high C-reactive protein concentration. Additionally, the levels of tumor markers, including carcinoembryonic antigen, carbohydrate antigen 19-9, neuron specific enolase and squamous cell carcinoma antigen, were within the normal limits. Computed tomography (CT) revealed a well-defined inhomogeneous mass in the right chest wall and the lesion showed a heterogeneous isointense signal on T1-weighted magnetic resonance imaging (MRI) and a high-intensity signal on T2-weighted MRI. The CT and MRI observations indicated the diagnosis of MFH or myxoid type liposarcoma. In addition, a percutaneous needle biopsy was performed and yielded three tissue fragments from the mass; grossly, the tissues appeared red and soft. The pathological slices was visualized using an Olympus BX31 microscope (Olympus, Tokyo, Japan) at ×100 and ×400 magnification. Microscopically, a storiform pattern of spindle cells and multinucleated giant cancer cells with atypical mitotic figures were observed, as well as hemorrhaging and necrosis. Furthermore, the immunohistochemical studies were negative for cytokeratin 5/6 and calretinin, but positive for vimentin (Fig. 1); therefore, MFH was diagnosed. Since the tumor was regarded as unresectable, RT was proposed to reduce the size of the tumor and ameliorate the symptoms associated with tumor growth. Epidermal growth factor receptor (EGFR) mutation tests were also performed to gain a better understanding. However, the sequencing analysis showed that exon 19 of the EGFR gene harbored a heterozygous in-frame deletion removing amino acids 746 to 750 (delE746-A750). Based on this result, gefitinib was prescribed at a dose of 250 mg/day as maintenance therapy.

### Radiosurgical planning

SBRT was performed using CyberKnife^®^ (Accuray Corporate, Sunnyvale, CA, USA) technology, which is a robotic, image-guided, full-body RT system. The system was equipped with the Synchrony^®^ Respiratory Tracking Device (Accuracy Corporate), which enables the CyberKnife^®^ technology to compensate for any tumor motion; however, the device requires the insertion of gold fiducial markers close to the tumor. Briefly, patients must have three gold fiducial markers (3×0.8*-*mm gold seeds) implanted inside the tumor to determine the tumor position. The fiducial markers may be placed by percutaneous needle placement using an 18-/19-gauge coaxial needle under image guidance and local anesthesia. Approximately one week following the placement of the fiducial markers, CT simulation was performed for the purpose of treatment planning. This time interval is sufficient to allow resolution of the edema and to identify any injury that may have occurred during the fiducial placement, as well as to allow for fiducial stabilization. In the present study, the MFH of the right chest wall measured 14.8×8.1 cm, with a 1,097-cm^3^ volume. The patient was subsequently prescribed an RT dose of 75 Gy to the 75% isodose line, which was delivered in five fractions. In addition, the volume of the right lung receiving ≥20 Gy was 15%.

### Clinical outcome

**Figure 2:**
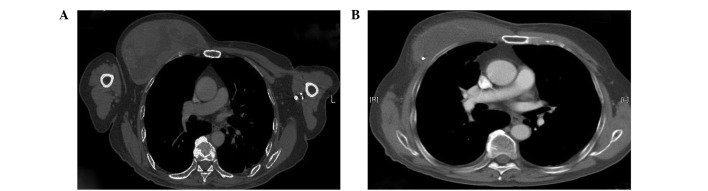
Computed tomography of the thorax showing (A) the tumour prior to CyberKnife radiotherapy and (B) the marked reduction in the tumour size following CyberKnife^®^ radiotherapy.

At four months following the CyberKnife^®^ RT, the tumor had reduced from 14.8×8.1 to 10.9×4.5 cm in size and notably, the solid lesion of the mass had significantly reduced (Fig. 2). The treatment-associated toxicity was assessed according to the Radiation Therapy Oncology Group (6) and the patient experienced grade 1 acute toxicity fatigue and mild radiodermatitis. Furthermore, late grade 1 toxicity radiodermatitis and pneumonitis were observed at four months following the CyberKnife^®^ RT.

## Discussion

MFH is the most common type of soft tissue sarcoma in adults accounting for 10% of all soft tissue sarcomas and predominantly occurs in males. Furthermore, MFH most commonly arises in the extremities (68%), trunk (16%) and retroperitoneum (16%), however, rarely presents in the chest wall (3). The majority of MFH tumors in adults can be classified according to a specific line of differentiation into the following five subtypes: Pleomorphic storiform, myxoid, giant cell, inflammatory and angiomatoid (7). The pleomorphic storiform and myxoid subtypes are generally high-grade neoplasms, while the others are usually low-grade sarcomas (8). The mean age of patients with primary MFH of the chest wall is 64.9 years (age range, 6–94 years) (2). Furthermore, the most common complaints are a non-painful mass (75%), a painful mass (13%) and pain alone (10%) (9). The present patient was older than the average MFH patient (77 years), and the patient’s initial presentation was a gradually increasing painless mass in the right chest wall, with intermittent fever. The features of the MFH identified by imaging of the chest wall were non-specific. The CT demonstrated a heterogeneous enhancing mass and the MRI showed signal intensities that were similar to, or lower than, that of muscle on T1-weighted images, which are often inhomogeneous and similar to, or greater than, that of adipose tissue on T2-weighted images (10).

MFH has a high propensity for local recurrence and distant metastasis; however, resection with negative microscopic margins decreases the incidence of local recurrence. Complete surgical resection at the time of primary tumor presentation remains the most effective therapy and additional chemotherapy may aid in the treatment of MFH (11,12). Mauri *et al* (13) also reported that the tyrosine kinase inhibitor may effectively function on patients affected by advanced stage MFH. In the current case, the EGFR mutation test was performed as a compassionate attempt. However, the sequencing analysis showed an in-frame deletion (delE746-A750) in exon 19 of the EGFR gene. Based on this result, gefitinib was administered at a dose of 250 mg/day and subsequently, the patient’s condition remained stable. RT has also been described as a definitive treatment for MFH patients with unresectable or positive surgical margins (14). The CyberKnife^®^ RT combined with respiratory gating in patients, as well as high total-dose RT with hypofractionation may be used to achieve permanent local control (5). In addition, Nagano *et al* (15) reported a patient with MFH presenting in the buccal region who underwent irradiation using a CyberKnife^®^ system following the failure of external RT at a dose of 38 Gy. The CyberKnife^®^ irradiation was performed twice at doses of 37 and 25 Gy. In the present case, the CyberKnife^®^ irradiation was delivered at a dose of 75 Gy in five fractions over five days. Following the treatment, the tumor gradually reduced in size with tolerable levels of toxicity.

In conclusion, the present study reports the feasibility of SBRT (specifically, CyberKnife^®^ RT) for the palliation of a patient with an MFH arising in the chest wall. No grade 3 or higher toxicity was observed in the patient, which indicates that this technique may be a useful treatment for unresectable MFH of the chest wall. However, further study is required to determine the optimal target dose, long-term toxicity and efficacy of this approach.
